# From a genome assembly to full regulatory network prediction: the case study of *Rhodotorula toruloides* putative Haa1-regulon

**DOI:** 10.1186/s12859-021-04312-3

**Published:** 2021-08-10

**Authors:** Jorge Oliveira, Miguel Antunes, Claudia P. Godinho, Miguel C. Teixeira, Isabel Sá-Correia, Pedro T. Monteiro

**Affiliations:** 1 INESC-ID, Lisbon, Portugal; 2 iBB - Institute for Bioengineering and Biosciences/ i4HB - Associate Laboratory Institute for Health and Bioeconomy, Instituto Superior Técnico, Universidade de Lisboa, Lisbon, Portugal; 3 Department of Bioengineering, Instituto Superior Técnico, Universidade de Lisboa, Lisbon, Portugal; 4 Department of Computer Science and Engineering, Instituto Superior Técnico (IST), Universidade de Lisboa, Lisbon, Portugal

## Abstract

Numerous genomes are sequenced and made available to the community through the NCBI portal. However, and, unlike what happens for gene function annotation, annotation of promoter sequences and the underlying prediction of regulatory associations is mostly unavailable, severely limiting the ability to interpret genome sequences in a functional genomics perspective. Here we present an approach where one can download a genome of interest from NCBI in the GenBank Flat File (.gbff) format and, with a minimum set of commands, have all the information parsed, organized and made available through the platform web interface. Also, the new genomes are compared with a given genome of reference in search of homologous genes, shared regulatory elements and predicted transcription associations. We present this approach within the context of Community YEASTRACT of the YEASTRACT + portal, thus benefiting from immediate access to all the comparative genomics queries offered in the YEASTRACT + portal. Besides the yeast community, other communities can install the platform independently, without any constraints. In this work, we exemplify the usefulness of the presented tool, within Community YEASTRACT, in constructing a dedicated database and analysing the genome of the highly promising oleaginous red yeast species *Rhodotorula toruloides* currently poorly studied at the genome and transcriptome levels and with limited genome editing tools. Regulatory prediction is based on the conservation of promoter sequences and available regulatory networks. The case-study examined is focused on the Haa1 transcription factor—a key regulator of yeast resistance to acetic acid, an important inhibitor of industrial bioconversion of lignocellulosic hydrolysates. The new tool described here led to the prediction of a RtHaa1 regulon with expected impact in the optimization of *R. toruloides* robustness for lignocellulosic and pectin-rich residue biorefinery processes.

**Supplementary Information:**

The online version contains supplementary material available at 10.1186/s12859-021-04312-3.

## Background

The analysis of newly sequenced genomes of new species and/or strains remain hindered by the lack of biological tools and databases, currently made available only for model organisms. Additionally, genome annotation is mostly limited to the prediction of gene function, providing no clues on the functionality of promoter sequences and derived gene expression regulation.

*cerevisiae*

Currently, dedicated genomic databases are available for the best studied yeasts, such as *Saccharomyces cerevisiae* (e.g. *Saccharomyces* Genome Database, SGD [1]), *Candida* species (*Candida* Genome Database, CGD [2]), among others, but rarely include data on promoter sequences and their impact on gene expression. YEASTRACT + (Yeast Search for Transcriptional Regulators And Consensus Tracking) + [3] is a portal for three distinct but interconnected databases focused on transcriptional regulation in yeasts: Yeastract, which focus on the model yeast and cell factory *Saccharomyces cerevisiae*; PathoYeastract, created to provide a resource for clinicians and biomedical scientists working with four pathogenic Candida species; N.C.Yeastract, created to guide the analysis and optimization of non-conventional biotechnologically-relevant yeasts. Like the aforementioned databases, YEASTRACT + provides not only Gene/Protein and orthology information but also curated regulatory information, including transcription factor binding sites, experimentally validated transcription factor-target gene associations and, more recently, cross-species regulatory network comparisons.

This paper describes a new tool for the systematic conversion of genomic sequences into organism focused databases that provide genome annotation at the gene and promoter levels. From a genome of interest from NCBI in the GenBank Flat File (.gbff) format, and with a minimum set of commands, the setup procedure enables to have all the information parsed, organized in a local database and provide a local web interface with similar tools to the ones available in the Yeastract database. This permits other communities to install the platform independently, without any constraints, thus benefiting from the experience gathered with the construction and continuous support of the YEASTRACT + portal.

This paper starts by explaining how the database is built and describing the required steps for starting a local instance of Community YEASTRACT and how to populate it with multiple layers of information, from the basic gene assembly to documented regulations, homology, synteny, potential regulations and Gene Ontology. Afterwards, in order to show-case the value of our tool in the context of Community YEASTRACT for knowledge inference, we present a case study on the lipid producing yeast cell factory *Rhodotorula toruloides.* This study extensively exploits the interconnectivity of the YEASTRACT databases to perform whole-genome promoter and TFBS conservation analysis and infer transcription regulatory networks. A specific *S. cerevisiae* acetic acid resistance regulator—Haa1, is used as an example to show the potential of this platform for the prediction of TFBS and documented regulatory associations analysis for *R. toruloides* Haa1 (RtHaa1) based on comparative genomics.

*Rhodotorula toruloides* is a nonpathogenic, red-colored basidiomycetous fungus. It is an oleaginous yeast that can accumulate lipids up to over 70% of its dry cell weight [4]. It is also a good producer of carotenoids and some important enzymes. *R. toruloides* can use a wide range of carbon sources for growth and is tolerant to inhibitory compounds found in biomass hydrolysates [5]. It is a good example of a biotechnologically relevant yeast, for which there is availability of genome assemblies from NCBI, but currently no database or tools for the comprehensive study of regulatory networks. The ability to predict, at the genomic scale, the regulatory interactions in *R. toruloides*, particularly those underlying the response to bioprocess related stresses, is expected to be useful to guide the selection and design of more robust strains for industrial applications. In particular, for the increase of *R. toruloides* acetic acid tolerance needed for the improved use of the carbon sources present in sugar beet pulp hydrolysates [6] for which the increased expression of RtHaa1 and the RtHaa1-regulon may be useful [7].

## Methods

Our approach relies on the use of a relational database [8] to store and organize all the genomic data, and a web server running PHP [9] as server side code to analyze and provide web functionalities to the end user.

The following sections thoroughly describe the considered relational database schema, and the procedure a given database administrator should follow to install and step up the our architecture on a personal computer and/or server.

### Source code

The complete source code for the front-end, back-end and database are freely available at https://gitlab.com/oliveira.jorge.88/web/. Each user can freely create its own instance of Community YEASTRACT and load his/her species of interest. Additionally, the code can be expanded and changed under the GNU General Public License. Error reporting and code optimizations are also welcomed. We provide a README.md file describing the installation requirements and the location of important configuration files. Also, a db_load/ directory contains the set of necessary scripts to perform all the pre-processing and loading of a given genome into the database. It also contains the scripts to perform all the post-processing tasks described in the next subsections, such as computing homology. Finally, the mysql.README.md file contains all the mysql commands to configure the database, to define the user access credentials and to load all the database structure.

Additionally, an instance of Community YEASTRACT is available at http://yeastract-plus.org/community/. The yeast species contained in this instance distinguish themself from the rest of the portal by not having regular data curation and update. Nonetheless, compared to a standalone instance of the code, it still carries the advantage of the interconnectivity with the curated species, and therefore, the regulatory inference capacity.

### Preparing the database

**Fig. 1:**
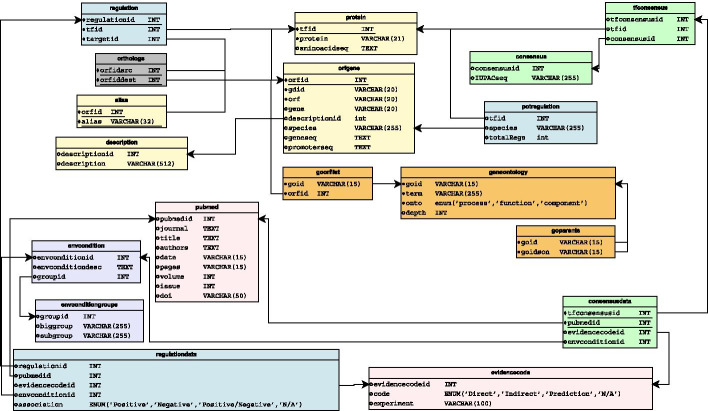
Relational database schema

The relational database is centered around the ORF/Gene concept (see Fig. 1). Here, each ORF/Gene as an associated species, the corresponding promoter and genomic sequences, chromosomal positions, and an external link to a reference database.

Then, each ORF/gene may have an associated protein with a given protein name and amino-acid sequence. Proteins in turn can have associated Transcription Factor Binding Sites (TFBSs) encoded as IUPAC sequences [10], together with the corresponding supporting reference. Additionally, each transcription factor may have a set of associated target genes, together with the corresponding supporting references.

Each supporting reference is composed by the associated PubMed ID [11], an environmental condition and an evidence code. This evidence code splits the experiments between four categories: DNA Binding evidence, Expression evidence, in silico Prediction, and not available. Environmental conditions are lumped together into 13 groups, each being sub-divided into sub-groups. To scale up the web functionalities, we filter by group/sub-group without having to parse the actual environmental conditions strings each query.

We also keep information about BLAST hits between ORF/genes from a given species against any other in the database (detailed in “Predicting a TF-network: the weak acid resistance regulator Haa1 as a case-study” section).

### Loading GenBank

The Genbank file format [12] is a standard file for genome sequence and annotation. Although the file has some flexibility for information, the essential information is always present and organized with pre-defined tags, thus allowing its retrieval for each sequence. This information is then validated and inserted in the database according to its schema (see Fig. 1). The genome of interest can be downloaded from NCBI, processed and loaded using the GBFF load tool (db_load/LoadGBFF.php).

### Computing orthologs/homologs

When no other information is available, databases like Candida Genome Database [2] indicate the BLAST Best-Hit for each gene. The BLAST Best-Hit gives a score for the alignment of a given ORF/Gene in species A against another ORF/Gene in species B. However, this information is not bidirectional, which means that the BLAST Best-Hit in one direction may not be present in the other direction.

Our orthology information starts with the same principle but has additional filters, as it follows a BLAST Reciprocal Best Score approach. The proteome of each species is used as the input for a BLASTp, using an E-value of 1e-5 and performed in a reciprocal way between all the species of the database. The BLAST hit with the highest score for each protein sequence is considered. Additionally, a tolerance of 10% relative to this highest score is applied, meaning that alignments with a score almost identical to the best are not lost. Only the gene pairs that are also the best score in the reverse BLAST (i.e. reciprocal) are considered. At this stage the homology is obtained.

This information is crossed with the synteny by comparing the genes adjacent to each homolog locus, where 15 neighbors are considered in each direction. Three levels of synteny “strength” were created, by requiring a minimum of 1, 2 or 3 neighbor genes in common to each pair of homologous genes.

### Loading documented data

A spreadsheet document is supplied to use as a standard form for documented data (db_load/CommunityDataSubmissionForm.xlsx). The first sheet of the document contains the data description and instructions. The second sheet defines the necessary fields to insert documented regulations and the third sheet defines the necessary fields to insert the transcription factor binding site information.

#### Loading documented regulations

In the second sheet, the required fields to maintain the database organization are: Transcription Factor, Target Gene, Strain, Supporting reference (PubMed ID), Evidence Code (DNA Binding, Expression, Prediction or N/A), Association Type (Direct, Indirect or N/A), Experimental Evidence, Environmental Condition, Environmental condition Group (e.g., Stress) and Environmental condition sub-group (e.g., Heat Shock).

Once filled, this form should be exported as a “tab-separated-values” file, and then used as an input for the upload tool (db_load/UploadRegulations.php). This tool performs a set of validations such as: the presence of all fields, the existence in the database of the transcription factor and target gene regulatory pair, among others. For each line in the form, if all the requirements are satisfied, the data is inserted in the database.

#### Loading transcription factor binding sites

In the third sheet, the required fields for the transcription factor binding site information are: Transcription Factor, Consensus, Strain, Supporting reference (PubMed ID), Evidence Code (DNA Binding, Expression, Prediction or N/A), Experimental Evidence, Environmental Condition, Environmental condition Group (*e.g.*, Stress) and Environmental condition sub-group (e.g., Heat Shock).

Similarly to the regulatory information, the binding site information should be exported as a “tab-separated-values” file, and then used as an input for the upload tool (db_load/UploadBinding.php), which will perform the necessary validations before inserting the data in the database.

Since the form only demands the PubMed ID as the unique identifier of the journal/article supporting the regulatory or binding site information, the database then needs to obtain the associated title, authors, journal, year, volume, etc., in order to appropriately display it in the web interface. This is also done in an automated way using the PubMed ID to extract from PubMed all the necessary information (using the JSON format of PubMed Entrez Programming Utilities, e.g. https://eutils.ncbi.nlm.nih.gov/entrez/eutils/esummary.fcgi?db=pubmed&id=31083555&retmode=json).

### Computing potential regulations

The database contains several functionalities which rely on the prediction of potential regulations, in other words, on performing the alignment of transcription factor binding sites on the promoter sequence of potential target genes. Although some of these functionalities perform this search on-the-fly as the user requests it, some functionalities such as “Rank based on unique TF binding sites in homologous genes” are computationally heavy and it would be impossible to perform all the alignments and present them in real-time, which calls for some pre-processing in order to provide a smooth response to the user.

This is achieved by pre-computing all the alignments between all TBFS from all species against the promoter sequence of all genes from all species (see potential regulation tool at db_load/computeHomoPotRegs.php). The database stores the name of the transcription factor and the name of the target genes, with the corresponding species, whenever at least one binding site, belonging to the transcription factor, has at least one occurrence in the promoter sequence of the target gene.

### Loading gene ontology

The Gene Ontology (GO) resource [13, 14] provides a computational representation of our current scientific knowledge about the functions of genes. In the proposed database structure (Figure 1) we include tables to load and represent all the terms of the three ontologies (molecular function, biological process and cellular component) as well as the hierarchy between terms (see db_load/GeneOntology.php). Also, we include a table to establish the relationship between each ORF/Gene and the associated GO terms. This last table must be loaded by a user-provided spreadsheet establishing the relationship between each ORF/Gene and GO term pair (see Gene Ontology load tool at db_load/insertGO.php).

## Case study: predicting transcriptional regulation in the lipid producing yeast *Rhodotorula toruloides*

Moving from a fossil-based industry to a sustainable bioeconomy demands the complete utilization of renewable feed-stocks and the efficient bioproduction of fuels and other specialty chemicals. *Rhodotorula toruloides* is one of the most promising yeast species for bioproduction from bioresidues [15]. In fact, the basidiomycete red yeast *R. toruloides* utilizes a wide range of sugars, has a particularly efficient natural metabolism of the acidic sugar D-galacturonic acid and of xylose, and is a producer of carotenoids, neutral lipids, and enzymes; all with important applications in the pharma and chemical industries [15]. Especially relevant is its natural ability to biosynthesize lipids that may be used as biofuels from lignocellulosic materials and pectin-rich residues with the potential to contribute to petroleum replacement [15, 16]. Although the genome sequence of *R. toruloides* NP11 is available [17], aspects of its peculiar metabolism were elucidated [18] and biological tools for genetic engineering were recently developed, their efficiency and exploitation is still limited [15]. For this reason, the improvement of the natural production of lipids and carotenoids by genetic engineering requires the capacity of predicting transcriptional regulation of metabolic genes, at a genomic scale, in *R. toruloides*. The possibility to make such predictions based on comparative genomics is an important and enormous challenge especially when yeast species are phylogenetically distant as it is the case of ascomycete and basidiomycete yeasts are involved. For all these reasons, the yeast species *R. toruloides* was selected as a case study to predict transcriptional regulation of a specific gene regulatory network described for other ascomycete yeast species. The possibility to do so is examined in the following sections.

### Whole-genome promoter conservation analysis

**Fig. 2:**
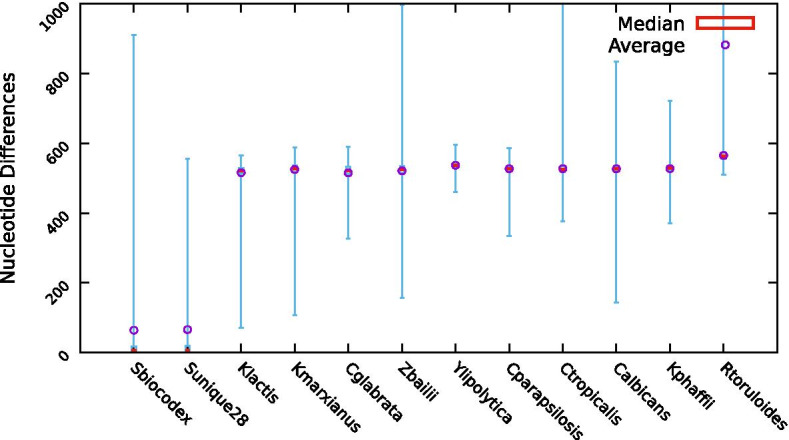
Promoter sequence nucleotide difference (using the Levenshtein distance), as a boxplot distribution, between *S. cerevisiae* S288C in comparison to all the species gathered in the YEASTRACT + platform, including the newly added *R. toruloides* NP11

As a first approach, the promoter sequences of homologous genes were compared at the genomic scale, using *S. cerevisiae* genes as the comparison basis, to all the species gathered in the YEASTRACT + platform, including the newly added *R. toruloides* NP11. Global nucleotide differences alignment was conducted using the Levenshtein distance, which allows for substitutions, insertions and deletions, and considering as promoter sequences the 1000 bp upstream of the START codon of each gene (Fig. 2).

Based on the obtained results, it is clear that promoter sequence conservation is relatively low, correlating to some extent with the overall phylogenetic distance of the considered species, the exception being *S. cerevisiae var. boulardii* biocodex and unique28 strains with an average of 65 nucleotide differences in the promoters of homologous genes. This is not surprising to see since *S. cerevisiae var. boulardii* strains and *S. cerevisiae S288c* are considered strains of the same species, as previously analysed [19]. When the species boundary is crossed the average variation increases immediately to over 500 differences per promoter, reaching nearly 600 differences per promoter in the more distant *R. toruloides*. As the species diverge, it is also possible to see that the dispersion of promoter variability increases towards higher levels. Indeed, in the two *Kluyveromyces* species, which, from the considered species, are the closest to *S. cerevisiae*, there are still many promoters of homologous genes with less than 500 nucleotide differences, suggesting a high degree of conservation among these promoters. At the other extreme, promoters of homologous genes in R. toruloides, in comparison to *S. cerevisiae*, never display less than 510 differences. These differences, in some cases, can go as high as 1000 nucleotides, corresponding to the full promoter. Although displaying lower levels of average nucleotide differences, promoter variability in *Z. bailii* IST302 and *C. tropicalis* MYA-3404 also reaches very high levels for some homologous gene pairs.

These results are indeed expected, as it has been seen that the degree of conservation of promoter sequences is much lower than that of coding sequences, which is hypothesized to be due to the non-functional nature of the majority of each promoter sequence [20].

### Whole-genome TFBS conservation analysis

In order to evaluate if transcription factor (TF) binding sites (TFBS) in the promoter sequence of homologous genes are more conserved than the remaining sections of the promoter in all the yeast species considered in YEASTRACT +, the *S. cerevisiae* TF binding sites were searched for in the promoters of homologous genes in the remaining species. Even though the alignment algorithm only allows for exact matches, the *S. cerevisiae* TFBSs deposited in YEASTRACT follow the IUPAC nucleotide code, which already encodes a certain level of redundancy. Therefore, we unfold each IUPAC sequence into several non-IUPAC sequences which are then considered for the alignment algorithm, keeping the noise and the false-positive rate as low as possible.

**Fig. 3:**
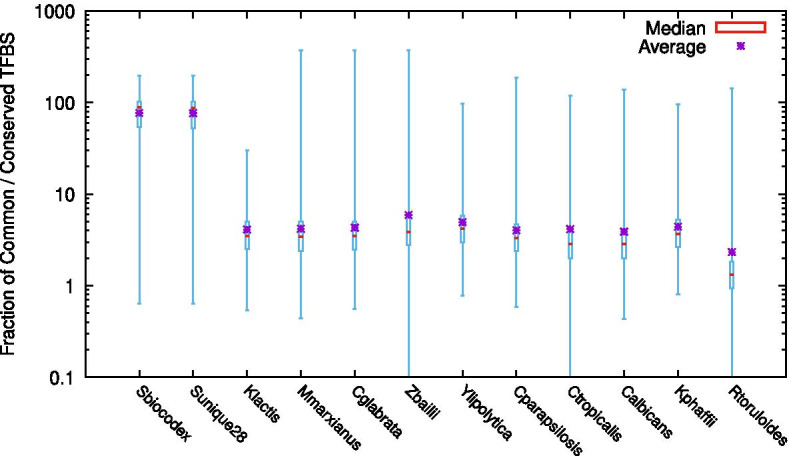
Fraction of common TF binding sites, relative to sites conserved only in *S. cerevisiae*, to all the species gathered in the YEASTRACT + platform, including the newly added *R. toruloides* NP11. For each species, the boxplot distribution of the fraction is presented

*S. cerevisiae* promoter regions include, on average, 42 distinct TF potential binding sites with the majority of the TFs predicted to bind to a gene promoter in *S. cerevisiae* S288C also predicted to bind to its homologue across all yeast species considered in YEASTRACT +. This observation is consistent with the fact that TF binding sites are predicted to evolve at a much slower rate, when compared to the remaining promoter sequence, and to be conserved among the closely related *Saccharomyces* species [20, 21]. To assess the conservation of TF binding sites, in Fig. 3, we show the distribution of the fraction of common TF binding sites relative to sites conserved only in *S. cerevisiae*, for all the species in YEASTRACT +.

It is observable that the fraction of shared TFBS relative to TFBS conserved only in *S. cerevisiae* between homologous genes in *S. cerevisiae* and *S. cerevisiae var. boulardii* is on average one order of magnitude higher than the remaining species. *R. toruloides* being the species sharing the least number of TFBS with *S. cerevisiae* (Fig. 3). Correlated to this are the observed promoter sequence variability and corresponding phylogenetic distance among the analyzed species. The smaller average number of TFBS in common between *R. toruloides* and *S. cerevisiae* relative to TFBS conserved only in *S. cerevisiae* highlights the high evolutionary distance between both relative to the remaining species. Besides, these differences suggest the presence of different motifs recognized by the transcription factors, divergence in gene regulation or even gain or loss of transcription regulators.

Altogether, this analysis suggests that the prediction of regulatory associations in each yeast species, based on the transcription factor binding sites characterized in *S. cerevisiae*, although possible, should be considered carefully, as it is only indicative, requiring experimental validation.

### Predicting a TF-network: the weak acid resistance regulator Haa1 as a case-study

Interestingly, both *R. toruloides* [5] and other oleaginous yeasts, such as *Cryptococcus curvatus*, *Rhodotorula glutinis*, *Lipomyces starkeyi* and *Yarrowia lipolytica* [22], are able to grow and produce high lipid concentrations from lignocellulosic hydrolysates, given that they are able to naturally use xylose as carbon source. However, the presence of inhibitory compounds in such hydrolysates, in particular acetic acid that is present due to the deacetylation of acetyl groups linked to the main chain of hemicelluloses, can affect yeast growth and metabolism depending on their concentrations and yeast strain tolerance [23]. For this reason, understanding the global yeast response to these inhibitors will pave the way for the development of superior strains for sustainable biorefinery processes. Given that yeast tolerance to acetic acid is strongly dependent on the transcription factor Haa1, predicting the Haa1-regulatory network is required to guide the increase of the robustness of poorly studied yeast species of biotechnological relevance.

The transcription factor Haa1 was first identified in *S. cerevisiae* as a determinant of acetic acid resistance [24] and its regulon characterized at the transcriptomic level [25]. The Haa1 specific binding site in target promoter regions was also defined [26], enabling the distinction between direct and indirect targets of its activity. Later on, *S. cerevisiae* Haa1 orthologs were characterized in the context of acetic acid tolerance studies in two other yeast species, the biotechnologically relevant *Zygosaccharomyces bailii* [7, 27] and the human pathogen *Candida glabrata* [28]. Given its importance in the context of industrial fermentation, Haa1 engineering has been attempted and proved successful in increasing *S. cerevisiae* acetic acid tolerance [29]. Its role is expected to be conserved in phylogenetically more divergent yeasts, such as *R. toruloides*, making Haa1 a fine candidate for transcription regulator engineering towards the construction of superior strains for lignocellulosic biorefineries, from a synthetic biology perspective.

Aiming the prediction of the Haa1 regulon in *R. toruloides*, two approaches were followed, based on previous knowledge on the Haa1 regulons in other yeasts, as deposited in the YEASTRACT + information system [3]: 1) a search for conserved Haa1 binding sites in homologous genes in *S. cerevisiae* and *R. toruloides* was evaluated; and 2) a search for conserved Haa1 target genes, with homologs in *R. toruloides*, across the three yeast species in which the TF-regulon is characterized. Since there is still no available transcriptional regulation data for *R. toruloides* or yeast from the Basidiomycota clade, the analysis of Haa1 regulons was made based only on yeast belonging to the Ascomycota clade (*S. cerevisiae*, *C. glabrata* and *Z. bailii*). Given the evolutionary distance among the considered yeast species, the reliability of the analysis might have been affected. Therefore, the predictions and conclusions taken from the following analysis, although useful to guide further studies, are only indicative, requiring experimental validation.

#### Based on TFBS conservation

Considering the experimentally determined *S. cerevisiae* Haa1(ScHaa1) binding sites [26], a search for Haa1 potential targets in *S. cerevisiae* S288C and *R. toruloides* NP11 was carried out. Haa1 recognition sequences in *S. cerevisiae* were identified in a two step process [26]. First, using electrophoretic mobility shift assay (EMSA) to evaluate the interaction of Haa1 with the promoter sequence of the target gene Tpo3, the consensus sequence GGCGAGGGG was identified. This recognition motif was, then, evaluated through Surface Plasmon Resonance (SPR), together with variations of the same sequence. Based on this analysis, four additional consensus sequences—GGCGAGAGG, GGCGCGGGG, GGCCAGGGG, AGCGAGGGG—were also found to be bound by Haa1, although showing slightly lower affinity to the protein. A minimal functional motif—SMGGSG—was then proposed following the analysis and combination of the aforementioned motifs [26].

If all the characterized consensus sequences for Haa1 are considered, including the minimal functional motif, the number of predicted targets includes 1952 common target genes in *S. cerevisiae* S288C and *R. toruloides* NP11, 2 unique predicted targets in *S. cerevisiae* and 1059 unique potential targets in *R. toruloides*. This number is much larger than the 86 documented Haa1 targets in *S. cerevisiae,* based on comparative transcriptomic analysis of the parental strain and an *HAA1* deletion mutant under acetic acid stress using DNA microarrays [25]. This suggests that there is a significant number of promoters harboring the Haa1 minimal consensus sequence but the binding of the transcription factor does not occur or could not be identified due to experimental limitations. It is reasonable to hypothesize that the experimentally validated Haa1 minimal consensus sequence is necessary but not sufficient for Haa1 to bind to its target promoters in the absence of the activating signal induced by acetic acid stress [30].

Considering only consensus sequences demonstrated to bind Haa1, there are 4 predicted targets in common to both *S. cerevisiae* and *R. toruloides*, 19 Haa1 potential targets unique to *S. cerevisiae*, and 669 unique to *R. toruloides*. Another hypothesis is that the Haa1 regulon may be much larger in *R. toruloides* than in *S. cerevisiae* or that the demonstrated binding site for *S.cerevisiae* does not coincide with its counterpart in *R toruloides*.

Altogether, this example illustrates that the ability to predict TF targets based on identified consensus sequences depends deeply on the degree of specificity of those sequences and on the experimental conditions under which the TF is biologically active. Indeed, whatever assumptions are made based on conservation of TF consensus sequences require a conservative approach and demand experimental validation and the consideration of post-transcription regulation events necessary to activate the TF binding activity.

#### Based on conserved documented regulatory associations

Aiming the prediction of the Haa1 regulon in *R. toruloides*, a second approach was attempted. The regulatory associations experimentally demonstrated for Haa1 transcription factors in three other yeast species were used to predict Haa1 regulated genes in this oleaginous yeast. This search was conducted for the *RHTO_01077* gene, encoding the closest homolog to the Haa1 protein in *R. toruloides*, using the “Search for genes” query in the *R. toruloides* web page created in the Community YEASTRACT database. In each case, the “Search for Homologous Regulations in:” option was used to select each one of the three species for which data on Haa1 regulated genes is available: *S. cerevisiae*, *C. glabrata* and *Z. bailii*.

**Fig. 4:**
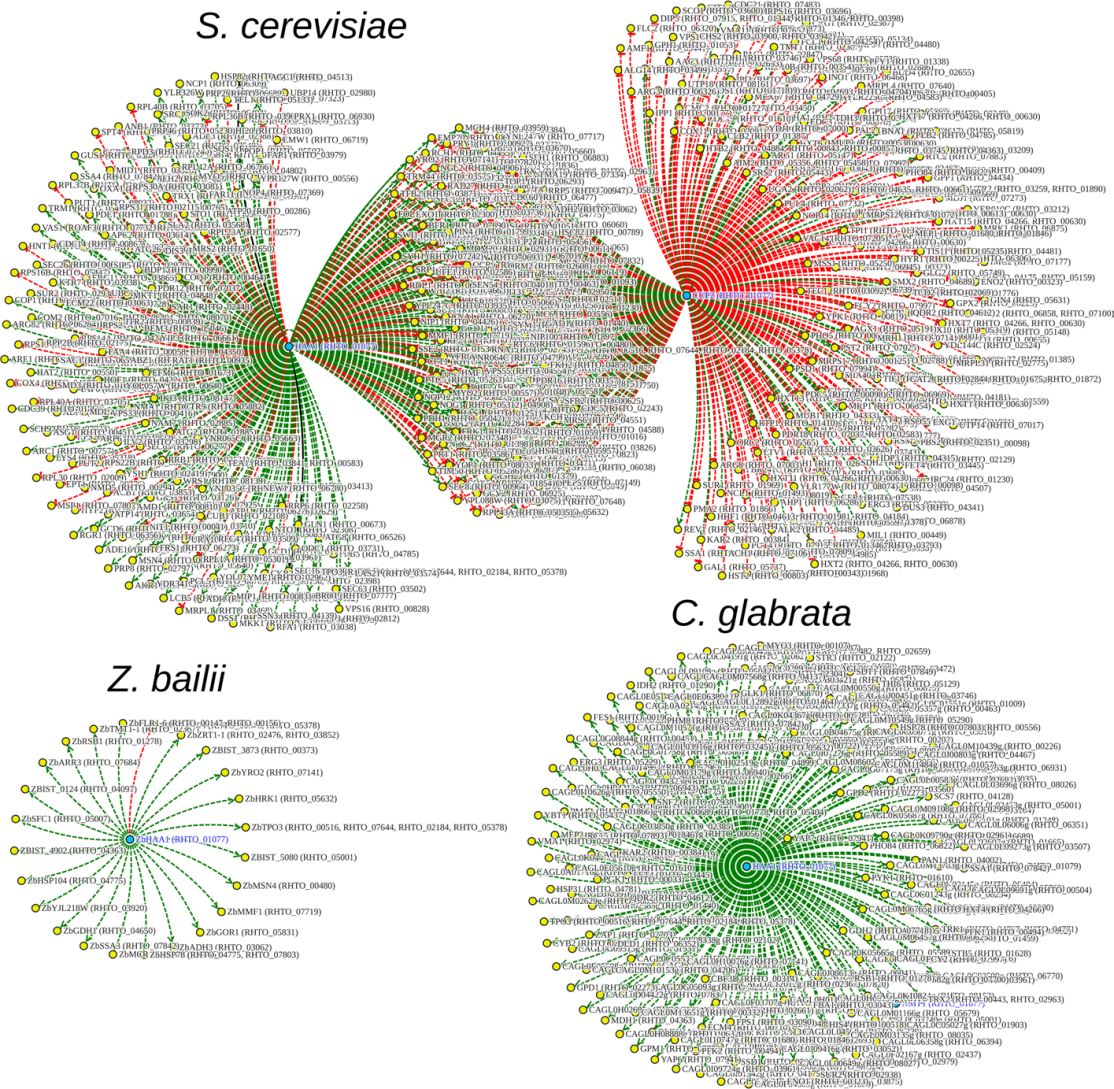
Predicted putative networks for the *RHTO_01077* gene—encoding the closest homolog of the Haa1 protein in *R. toruloides*—projected from the documented regulatory associations of *S. cerevisiae*, *C. glabrata* and *Z. bailii*

According to the obtained results, RtHaa1 targets in *R. toruloides* may be predicted from ZbHaa1 targets in *Z. bailii*, CgHaa1 targets in *C. glabrata* and from ScHaa1 and ScAce1 targets in *S. cerevisiae* (Fig. 4). The duplicity of results in *S. cerevisiae* derives from the fact that both ScHaa1 and ScAce1 are close homologs of RtHaa1, ScHaa1 being only slightly more closely related to RtHaa1 than ScAce1.

Using *S. cerevisiae* as the basis for target prediction in *R. toruloides*, RtHaa1 is predicted to have 578 target genes, while based on the data available for *C. glabrata* and *Z. bailii* only 214 and 28 targets may be predicted for RtHaa1, respectively. The difference in the number of Haa1 targets experimentally demonstrated for each species is likely to reflect the amount of available published datasets, making *S. cerevisiae* the most informative and, as such, at least in this particular case, the most promising species to enable regulatory network predictions. Nonetheless, and since the targets of homologous transcription factors are found to vary, at least partially, from organism to organism. Cross-species prediction is a promising tool mainly to enable the prediction of the core regulon of each transcription factor; likely to be the most conserved across evolution.

**Fig. 5:**
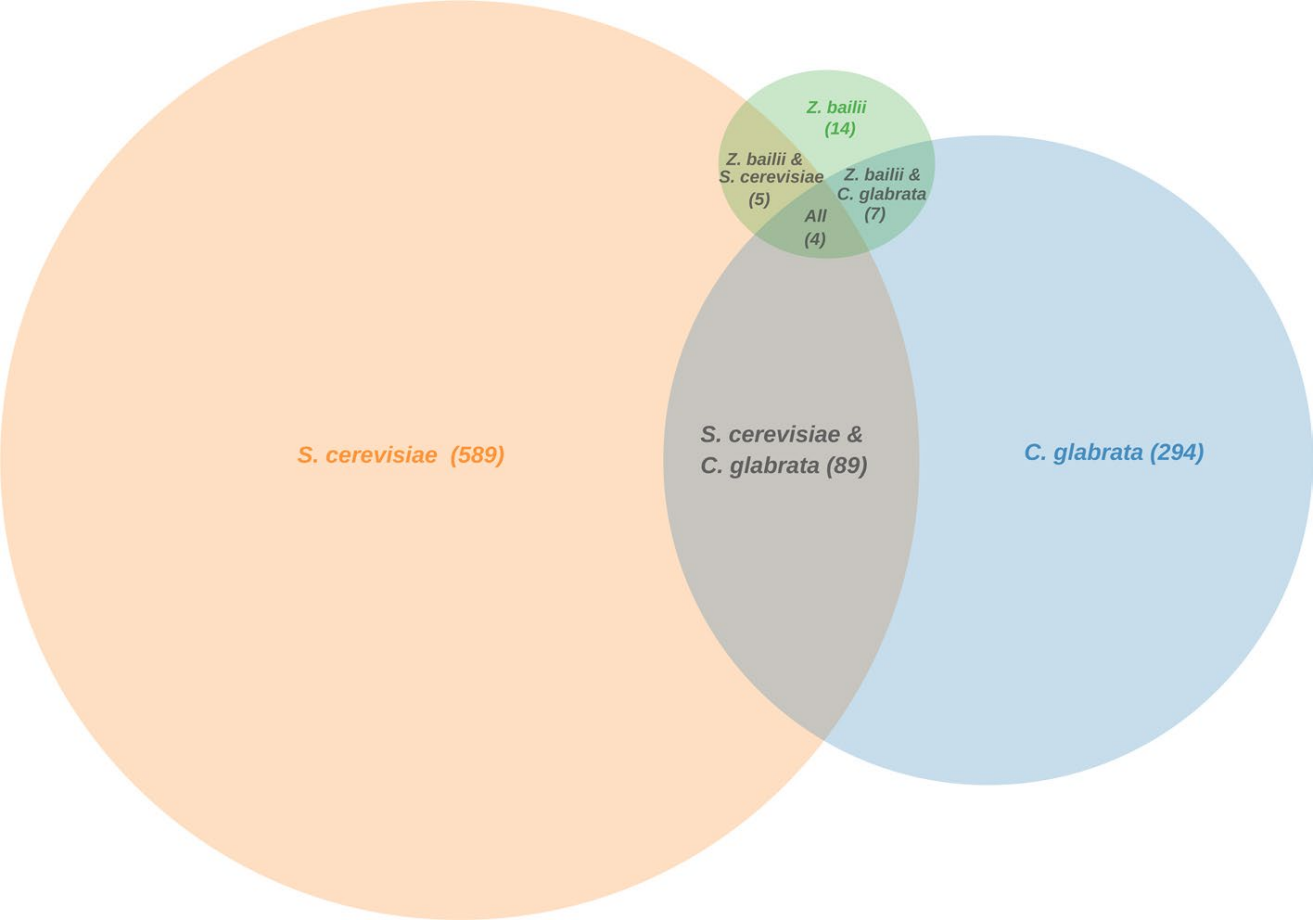
Inferred Regulon Venn Diagram, of the Haa1p transcription factor targets, considering the documented regulatory associations in *S. cerevisiae, C. glabrata* and *Z. bailii*

By intersecting the Haa1 regulons from *S. cerevisiae*, *C. glabrata* and *Z. bailii*, a small set of 11 genes is found to be the core regulon of this transcription factor (Fig. 5). By the time of this analysis, the core regulon included four *TPO2/3* homologs (*RHTO_00516*, *RHTO_07644*, *RHTO_02184* and *RHTO_05378*), encoding plasma membrane transporters of the Major Facilitator Superfamily (MFS) proposed to mediate acetate efflux when yeast cells are cultivated in the presence of acetic acid [24]. Another core gene of the Haa1 regulon was *RHTO_05632*, homologous to *S. cerevisiae* *HRK1*, a well-documented Haa1 target gene encoding an Npr1/Hal5 kinase found to mediate the phosphorylation of several membrane-associated acetic acid-responsive proteins [25, 31]. Also, we identified *ScYRO2* homolog (*RHTO_07141*), encoding a poorly characterized determinant of acetic acid tolerance [32] and *HSP104* (*RHTO_04775*) and *SSA4* (*RHTO_07842*), which are very important stress responsive genes, required for weak acid stress response and tolerance in *S. cerevisiae* [33, 34]. Two of the remaining genes identified, *MDH1* (*RHTO_04363*) and *TMT1* (*RHTO_02367*), encode enzymes that control the flux through the TCA cycle, a central pathway that controls the balance between energy generation and key anabolic processes [35, 36], and *RHTO_03062* which is homologous to the ADH genes, encoding alcohol dehydrogenases in *S. cerevisiae*.

Since the number of Haa1 target genes found in *Z. bailii* is comparatively small compared with the two other species, focus was given to the Haa1 targets shared between *S. cerevisiae* and *C. glabrata* with homologs in *R. toruloides*. These include a list of 69 genes (Additional file 1: Table S1), whose functional categories were analysed using the GO enrichment analysis tool Panther (http://pantherdb.org/webservices/go/overrep.jsp). The functions that stand out are mostly related to transport, including: “ion transport”; “carbohydrate transport”; and “xenobiotic detoxification by transmembrane export across the plasma membrane”. These are indisputably highly relevant for weak acid stress tolerance, as they include genes such as *PMA1* and* PMA2*, encoding two isoforms of yeast plasma membrane H + -pump ATPase, and those encoding polyamine transporters of the Major Facilitator Superfamily that also have been proposed to catalyse the extrusion of acetate, Tpo2 and Tpo3 [24].

**Fig. 6:**
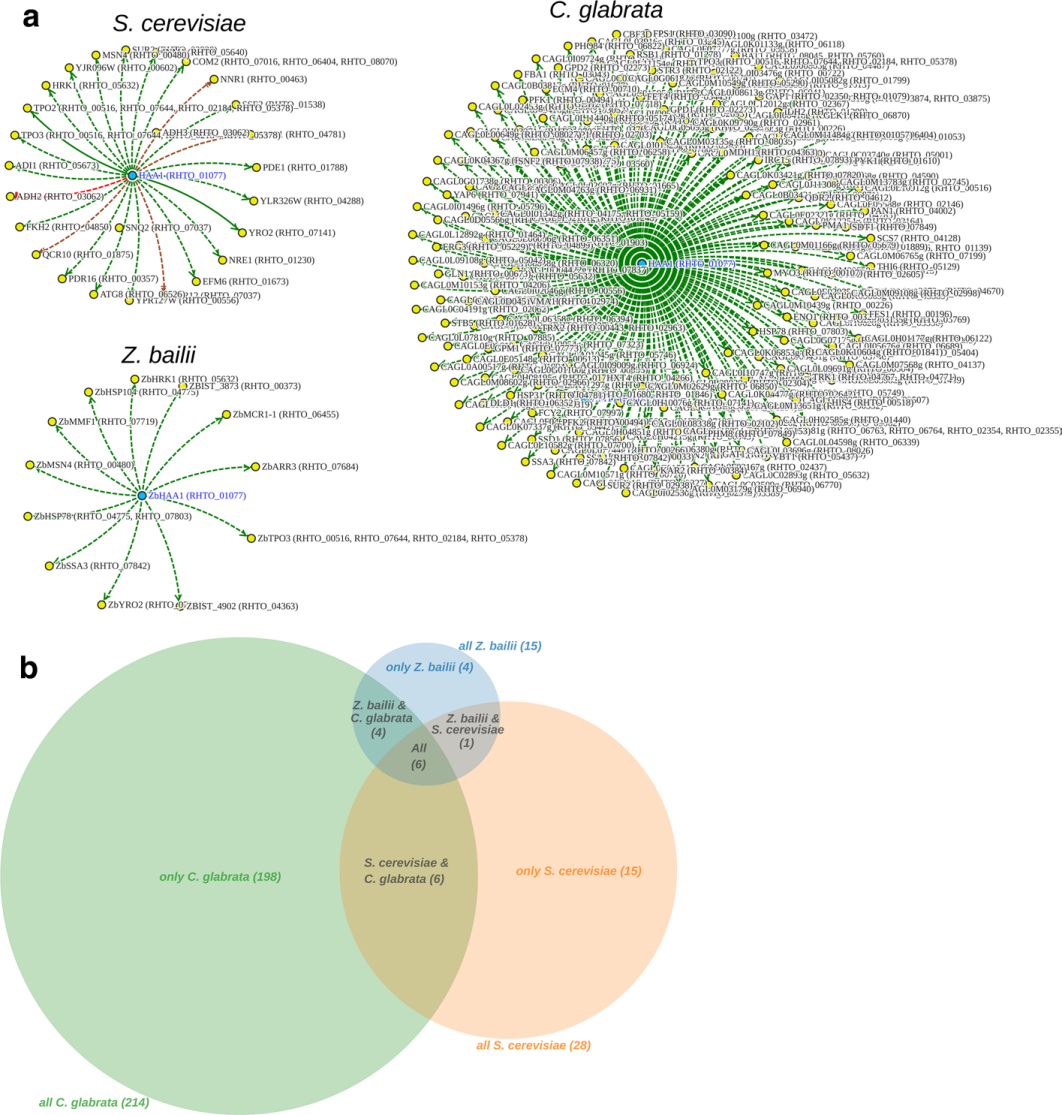
The RtHaa1 regulon in weak acid stress. **A** Predicted RtHaa1-regulon, based on the set of documented regulatory associations of *S. cerevisiae*, *C. glabrata* and *Z. bailii* under weak acid stress. **B** Inferred Regulon Venn Diagram, of the Haa1 transcription factor targets, considering the documented regulatory associations in *S. cerevisiae*, *C. glabrata* and *Z. bailii* under weak acid stress conditions

Further exploiting the potential of YEASTRACT + tools, the networks obtained in the “Search for Genes” tool can be filtered in-place to show regulatory associations only associated with a given environmental condition of interest. Taking our previous example, the filtering of the Haa1-regulon to the environmental condition “Weak acid stress” resulted in three networks displaying the genes predicted to be regulated by RtHaa1 considering the *R. toruloides* homologs from the documented Haa1 targets in *S. cerevisiae*, *C. glabrata* and *Z. bailii* in this environmental condition (Fig. 6A). It is important to notice that although the prediction based on the regulations documented for *S. cerevisiae* includes a lower number of target genes, it is based on several expression studies, whereas the information regarding the network based on *C. glabrata* and *Z. bailii* arise from only one study each.

Considering the intersection of these three datasets comprising the Haa1-target genes documented in the presence of weak acid stress, 6 *R. toruloides* genes are found to be the core regulon of this transcription factor under this environmental condition (Fig. 6B): four *TPO2/3* homologs (*RHTO_00516*, *RHTO_07644*, *RHTO_02184* and *RHTO_05378*), one *HRK1* homolog (*RHTO_05632*) and one *YRO2* homolog (*RHTO_07141*).

In summary, the data available at the YEASTRACT + platform for Haa1 transcription factors in several yeast species can be used to extrapolate what the RtHaa1 regulon may be. The prediction may be less conservative, by considering that the homologs of all the Haa1 targets in all species may also be Haa1 targets in *R. toruloides* under acetic acid stress. However, the use of additional filters, such as exemplified herein (e.g. considering only Haa1 targets shared by 2 or more species, or considering only targets activated in particular environmental conditions), are recommended and may be exploited at the discretion of the user to reach more reliable predictions, all of which still require experimental validation but may prove invaluable in guiding this research work.

## Conclusion

In this study, a tool for the analysis of genome sequences, from their NCBI deposition format into comprehensive databases, is offered. This tool includes a pioneering approach to the subject of genome annotation by the inclusion of functional promoter analysis, based on the evaluation of transcription factor consensus occurrence and regulatory network prediction based on the corresponding knowledge gathered for related species. The offered tool was primarily designed for yeast species, taking advantage of the support given by the YEASTRACT + portal and the data included therein. It is provided in the context of the Community YEASTRACT database, but it may also be installed as an independent platform and applied to other organisms.

The usefulness of the presented tool was exemplified with the analysis of the genome of the oleaginous yeast cell factory *R. toruloides*. A dedicated database for this yeast was created, including gene and promoter annotation data. Regulatory prediction based on the conservation of promoter sequences and regulatory networks was evaluated. Gene regulation inference may be conducted by the search for transcription factor consensus sequences, which are conserved to some extent in the promoters of homologous genes. More reliable predictions may, nonetheless, be extrapolated from regulatory associations documented for homologous genes in closely related species. The limitation relies on the lack of suitable and trustable biological information available. The case-study examined in this study, concerning the prediction of the Haa1 regulon in *R. toruloides* (RtHaa1) in response to acetic acid stress is paradigmatic of both the potential of the tool and the limitations that this approach still has due to the limiting biological data available to support and allow robust predictions. However, the exploitation of this tool was able to lead to the proposal of a putative RtHaa1 regulon that makes sense from the biological point of view. This outcome paves the way to the prediction, at the genomic scale, of the regulatory interactions occurring in *R. toruloides*, particularly those underlying the response to acetic acid stress, that are expected to be useful to guide the selection and design of more robust strains for lignocellulosic biorefineries.

## Supplementary Information

**Additional file 1. List of genes whose functional categories were analysed using GO enrichment.**

## Data Availability

All the code and step-by-step instructions to download, pre-process, and load a given genome of interest during the current study are available in the GitLab repository, https://gitlab.com/oliveira.jorge.88/web.

## Abbreviations

TF: Transcription factor
TFBS: Transcription factor binding site
GO: Gene ontology
